# A novel likely pathogenic variant in the *UMOD* gene in a family with autosomal dominant tubulointerstitial kidney disease: a case report

**DOI:** 10.1186/s12882-020-02022-1

**Published:** 2020-08-26

**Authors:** Ying Wang, Haibo Liu, Qingnan He, Zhuwen Yi, Yongzhen Li, Xiqiang Dang

**Affiliations:** 1 Department of Pediatrics, the Second Xiangya Hospital, Central South University, 139 Renmin Road, Changsha, 410011 Hunan China; 2 Laboratory of Pediatric Nephrology, Institute of Pediatrics, Central South University, Changsha, Huan China

**Keywords:** Hyperuricaemia, Kidney disease, ADTKD, *UMOD*

## Abstract

**Background:**

Autosomal dominant tubulointerstitial kidney disease (ADTKD) caused by a pathogenic variant in *UMOD* (ADTKD-*UMOD*) is a rare group of diseases characterized by hyperuricaemia with decreased urinary excretion of urate, gout and progressive chronic kidney disease. The mundane clinical characteristics often result in a failure to diagnose ADTKD-*UMOD*.

**Case presentation:**

In this report, we describe a 12-year-old boy who presented with polyarthritis, hyperuricaemia and tophi with a family history of 8 affected individuals. Clinical data, blood and urine samples of 3 affected members and 8 unaffected members were collected. Genetic testing of the eight genes (*UMOD*, *HPRT1*, *PRPS1*, *MTHFR*, *REN*, *HNF1b*, *URAT1* and *G6PC)* was performed using Sanger sequencing. A heterozygous missense variant (c.674C > G; p.T225R) in *UMOD* was found in this boy, his older brother with the same phenotype and his mother with hyperuricaemia, gout and chronic kidney disease.

**Conclusion:**

This case highlights the importance of family history and genetic testing for definite diagnosis. This novel variant extends the spectrum of known *UMOD* gene variants and further supports the allelic heterogeneity of ADTKD-*UMOD*.

## Background

Autosomal dominant tubulointerstitial kidney disease (ADTKD) is a rare underdiagnosed cause of end-stage renal disease (ESRD) that was proposed by KDIGO in 2015 [1–4]. It is caused by pathogenic variants in at least 5 different genes: uromodulin (*UMOD*), mucin 1 (*MUC1*), hepatocyte nuclear factor 1 beta (*HNF1B*), renin (*REN*), and the alpha subunit of the endoplasmic reticular membrane translocon (*SEC61A1*) and have been categorized as ADTKD-*UMOD*, ADTKD-*MUC1*, ADTKD–*REN*, ADTKD-*HNF1B*, ADTKD-*SEC61A1* or ADTKD-NOS (not otherwise specified), respectively [1–5]. There is no evidence to establish the prevalence of the different types of ADTKD, but ADTKD-*UMOD* and ADTKD-*MUC1* are the most frequently identified forms [1, 2]. Furthermore, among factors differentiating the different forms, hyperuricaemia and gout are more frequent in individuals with ADTKD-*UMOD* [1, 2]. ADTKD-*UMOD* was previously named UKD (uromodulin kidney disease), UAKD (uromodulin-associated kidney disease), FJHN (familial juvenile hyperuricaemic nephropathy) and MCKD2 (medullary cystic kidney disease type 2) [2] and is characterized by early-onset hyperuricaemia and gout caused by inappropriately decreased fractional urate excretion, bland urine sediment with absent-to-mild proteinuria, and the development of insidious renal failure with tubulointerstitial disease [1–3]. Patients usually develop ESRD between the ages of 20 years and 80 years, with most individuals requiring renal replacement therapy between the ages of 30 years and 50 years [2]. Some patients have medullary renal cysts [2, 4].

Here, we report a case of a boy with gouty arthritis who belonged to an ADTKD-*UMOD* family with 8 affected individuals (4 alive) from China and identified a novel likely pathogenic *UMOD* variant for this disease.

## Case presentation

A 12-year-old Chinese boy complained of swelling and pain of the right 1st metatarsophalangeal joint combined with tophi at age 10, and then arthritis affected the bilateral ankle. Therefore, he went to our hospital.

**Fig. 1:**
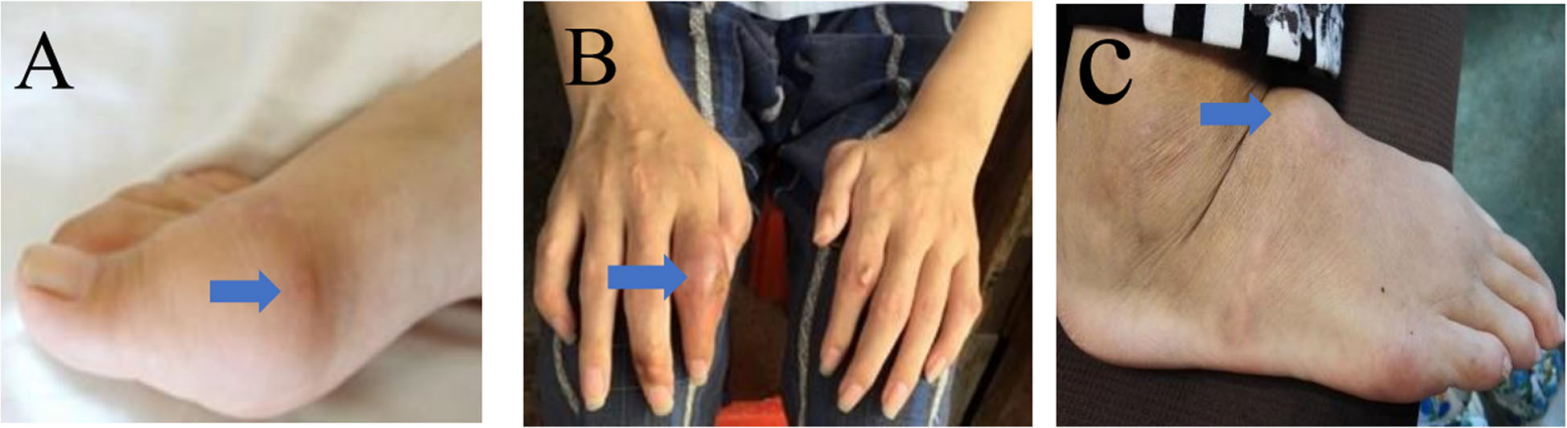
Photographs of tophi. Gout tophi were found in the proband (**a**), his elderly brother (**b**), and his mother (**c**)

**Fig. 2:**
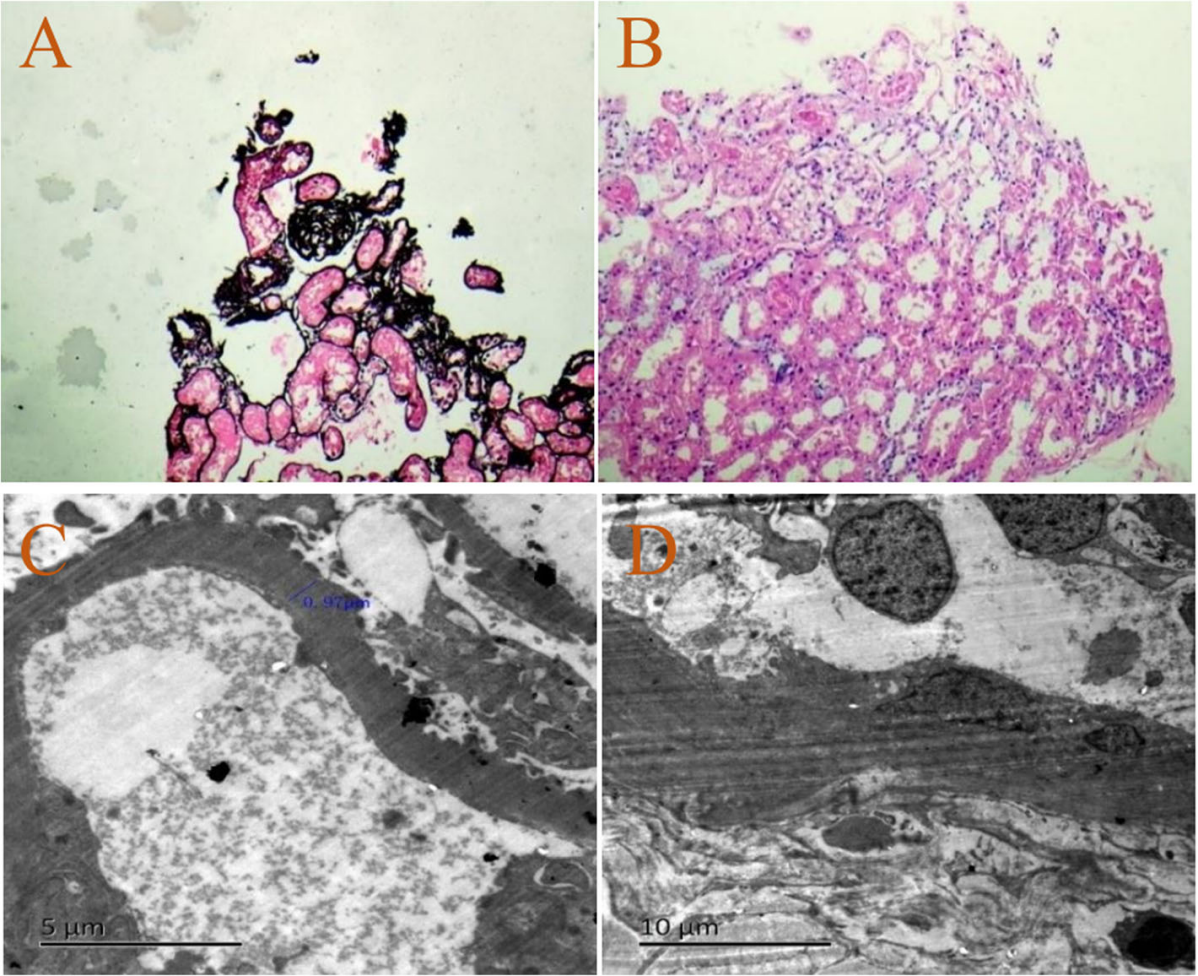
Light microscopy and electron microscopy of the proband’s renal biopsy. **a** Glomerulosclerosis (PASM, × 200). **b** Tubular atrophy (HE, × 200). **c** Basement membrane thickening. **d** Interstitial fibrosis and inflammatory cell infiltration

**Fig. 3:**
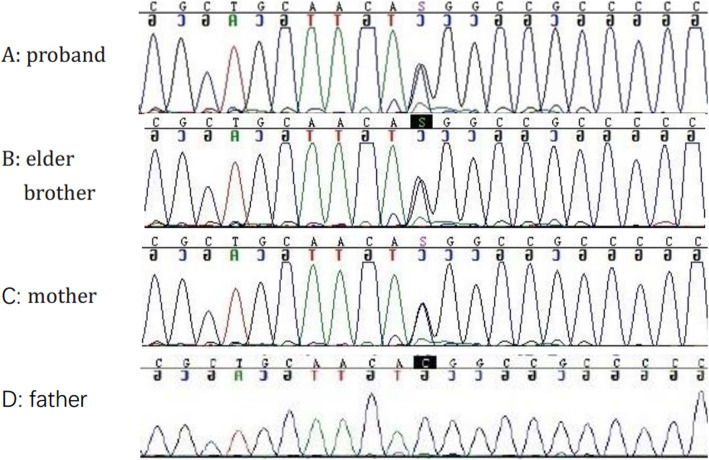
Genetic features of the study subjects. Genetic features of the study subjects. **a**-**c** Sequences of three affected individuals: the proband, the proband’s older brother, and their mother. **d** Sequence of an unaffected individual (the proband’s father)

The physical examination was unremarkable, except for tenderness, redness and swelling in the right 1st metatarsophalangeal joint and bilateral ankle with tophi (Fig. 1a). Laboratory data showed serum creatinine 61.7 μmol/L (reference range 50-80 μmol/L) and blood urea nitrogen 5.65 mmol/L (reference range 2.9–7.14 mmol/L). Laboratory tests revealed hyperuricaemia 461.4 μmol/L (serum uric acid reference range 155–357 μmol/L) and decreased excretion of UA (24 h urine UA 0.51 mmol, urinary UA excretion: 0.109 mg·kg^− 1^·h^− 1^, urinary UA clearance: 1.22 ml/min per 1.73m^2^, fractional excretion of UA: 1.1%). Normal or negative blood pressure, routine urinalysis, serum complements, ANA, anti-dsDNA, ANCA, rheumatoid factor, electrolytes and ultrasound of kidneys. Feet CT shows multiple bone destruction and bony defects of the ankles and feet, as well as soft tissue swelling. Light microscopy of renal biopsy specimens showed that one-in-six glomeruli had global sclerosis and extensive tubular atrophy (Fig. 2a, b). Electron microscope analysis showed vacuolar and granular degeneration of renal tubules, segmental basement membrane thickening (500–1000 nm), interstitial fibrosis, inflammatory cell infiltration and foot process fusion (Fig. 2c, d). Direct immunofluorescence staining was negative for immunoglobulin and complement. In the family history, the proband’s older brother was diagnosed with gout at age 17 (Fig. 1b), his mother was diagnosed with gout with stage 4 CKD at age 38 (Fig. 1c), his cousin was diagnosed with gout at age 28, his uncle died of gout and CKD at age 33, his maternal grandfather and two brothers of his maternal grandfather died of similar diseases when they were in their mid-forties. All of them had no other chronic disease. All participants in the study gave their informed consent. Clinical data, blood and urine samples of 3 affected members and 8 unaffected members were collected. Genetic testing of eight genes associated with hyperuricaemia (*UMOD, HPRT1*, *PRPS1*, *MTHFR*, *REN*, *HNF1b*, *URAT1* and *G6PC*) revealed that the 3 affected members carried a novel heterozygous missense variant (c.674C > G, p.T225R) in the *UMOD* gene (Fig. 3). In contrast, this likely pathogenic variant was not found in 8 unaffected members.

Finally, we diagnosed the three patients with ADTKD-*UMOD*. The proband and his elder brother were treated with allopurinol (100 mg/day) and colchicine (0.5 mg/day). However, several months later, they both discontinued regular therapy after the frequency of gouty flares was reduced for financial reasons. The two brothers have not developed ESRD to date, but their mother has received haemodialysis since March 2018.

## Discussion and conclusion

ADTKD-*UMOD* is different from primary gout, which is caused by abnormal metabolism of purine and/or poor UA excretion [6]. It is a known subtype of ADTKD and is characterized by a family history with autosomal dominant inheritance. Kidney Disease Improving Global Outcomes (KDIGO) guidelines recommend a diagnosis of ADTKD-*UMOD* either if the family history is compatible with autosomal dominant inheritance of CKD fulfilling the clinical characteristics, compatible histology in at least one affected family member or demonstration of a mutation in *UMOD* genes in an affected individual [2]. Lack of distinctive clinical features and incomplete acquisition of family histories often result in a failure to diagnose ADTKD-*UMOD* [3, 4]. Here, we report a family with 8 ADTKD-*UMOD*-affected individuals.

*UMOD* (OMIM 16p12.3 and 191,845) is composed of 11 exons, the first of which is noncoding. *UMOD* encodes uromodulin, which is produced in the epithelial cells lining the thick ascending limb (TAL) of Henle’s loop and the initial part of the distal convoluted tubule (DCT) [2, 4]. Uromodulin contains an N-terminal signal peptide that contains three epidermal growth factor (EGF)-like domains, an eight cysteine-rich domain (D8C) that is highly conserved in uromodulin homologues from different species, a zona pellucida-like (ZP) domain and a glycosylphosphatidylinositol (GPI) anchor segment in the C-terminal region [7]. Although uromodulin is the most abundant protein in normal urine and has multiple roles in kidney physiology, how pathogenic *UMOD* variants influence the reduced excretion of UA and cause renal failure is not completely understood [8].

The disease mechanism in ADTKD-*UMOD* probably includes the accumulation of mutant uromodulin in the endoplasmic reticulum of TAL cells, with secondary decreased cellular release and urinary excretion of wild-type protein [2]. Mutant uromodulin inhibits trafficking and activity of NKCC2 in TAL. The resulting defect in urinary concentration and consequent mild volume depletion can secondarily increase the reabsorption of UA in the proximal tubule as a possible cause of hyperuricaemia [2, 4, 9].

It has been shown that renal insufficiency is due to the accumulation of mutant uromodulin in the endoplasmic reticulum (ER), resulting in tubulointerstitial damage through ER stress and reduced urinary uromodulin excretion [3, 10]. Ryu ES et al. published that hyperuricaemia transforms renal tubule cells into fibroblasts and activates the renin–angiotensin system, leading to endothelial cell dysfunction [11]. The renal pathologic findings are interstitial fibrosis, inflammatory cell infiltrate, tubular atrophy, thickening and splitting of tubular basement membranes and normal glomeruli [1, 2, 4, 12]. Immunofluorescence for complement and immunoglobulins are negative [12].

Pathogenic variants are mainly localized to exons 3 and 4, which encode the three EGF-like domains and D8C [8, 9, 12, 13]. There is no “hot spot” [14]. In this family, we found the likely pathogenic variant p.T225R in exon 3 of *UMOD*, which has not been described previously, although Dahan et al. and Wolf et al. have reported missense variants at the same pathogenic variant site, p.T225M and p.T225k in 2003, respectively [15, 16]. The likely pathogenic variant was not found in the other 8 unaffected family members. His cousin has not undergone genetic testing for personal reasons. Threonine 225 is located between the first and the second cysteine residue pairs in D8C [17]. This variant is categorized as probably damaging, deleterious and disease causing by PolyPhen-2/PROVENAN/MutationTaster. This variant have been classified as “likely pathogenic” according to ACMG combining criteria with two instances of moderate evidence (PM2, PM5) and two instances of supporting evidence (PP1, PP3) of pathogenicity [18]. Although the function of D8C is unknown, it is believed to be crucial for maintaining the correct protein conformational structures of uromodulin. We presume that this likely pathogenic variant may lead to protein misfolding and defective trafficking and then generate inflammatory responses [10, 13].

Treatment options for ADTKD-*UMOD* are limited so far. Allopurinol or febuxostat (when allopurinol cannot be tolerated) is suggested as the drug for the treatment of hyperuricaemia and gout after the first attack of gout has resolved [2, 4, 19]. Controversy exists as to whether allopurinol ameliorates the progression of renal damage. A strict low purine diet is not known to be beneficial in hyperuricaemic patients with *UMOD* pathogenic variants [2]. Note that diuretics should be used with caution, and a low-salt diet is not recommended because they may aggravate hyperuricaemia [2]. The affected family members need annual testing of their kidney function, and kidney transplantation is the preferred choice for kidney failure, as there is no evidence that ADTKD-*UMOD* can recur after transplantation [2, 4].

In summary, we detected a novel likely pathogenic *UMOD* variant located within D8C in a single kindred with ADTKD-*UMOD*, which extends the spectrum of known *UMOD* gene variants and further supports allelic heterogeneity. Physicians should suspect ADTKD-*UMOD* in patients who present hyperuricaemia and chronic kidney disease with a family history, and genetic testing should be performed to confirm the diagnosis.

## Data Availability

The datasets analysed during the current study are available in the Genome Sequence Archive in National Genomics Data Center, Beijing Institute of Genomics, Chinese Academy of Sciences. The accession number is HRA000269. Please access it from the following link: https://bigd.big.ac.cn/gsa-human/browse/HRA000269 .

## Abbreviations

ADTKD: Autosomal dominant tubulointerstitial kidney disease
PCR: Polymerase chain reaction
PASM: Periodic acid-silver metheramine
PAS: Periodic acid Schiff
HE: Haematoxylin-eosin
CKD: Chronic kidney disease
UA: Uric acid
